# Flexible Silanized TiO_2_/SBS Nanocomposite Films with Optimized Emissivity for Passive Radiative Cooling Applications

**DOI:** 10.1021/acsaom.6c00402

**Published:** 2026-07-31

**Authors:** Martina Mercurio, Daniele Ceneda, Agostino Occhicone, Ilaria Fratoddi, Isabella Chiarotto, Marco Centini, Maria Cristina Larciprete

**Affiliations:** † Department of Basic and Applied Sciences for Engineering, Sapienza University, Via Antonio Scarpa, 14, 00161 Rome, Italy; ‡ Department of Chemistry, Sapienza University, P.le Aldo Moro 5, 00185 Rome, Italy

**Keywords:** radiative cooling, sustainable coating, passive thermal management, infrared radiation manipulation, freestanding nanocomposite films, silanized TiO_2_ nanoparticles

## Abstract

Passive radiative cooling (RC) requires materials that combine high solar reflectance with strong long wavelength infrared (LWIR) emissivity in the 8–13 μm atmospheric window (AW). In this work, freestanding nanocomposite films were fabricated by embedding MPTMS-functionalized TiO_2_ nanoparticles (TiO_2_NPs–MPTMS) into a styrene–butadiene–styrene (SBS) triblock copolymer matrix. The resulting composites, referred to as NPs-SBS, were designed to be flexible, scalable, and cost-effective for potential RC applications. The SBS polymer provides emissive response in the 8–13 μm AW, whereas the inorganic component governs the solar reflection properties. Surface functionalization yielded Ti–O–Si linkages and a silica-like shell that enhances nanoparticle dispersion and stability while introducing additional vibrational modes in the LWIR range. Field-emission scanning electron microscopy and energy-dispersive X-ray spectroscopy characterization confirmed grain-like aggregates composed of nanoparticles with diameters ranging between 30 and 60 nm and verified the surface functionalization. To optimize RC performance, composites containing 1, 5, and 7 wt % of TiO_2_NPs–MPTMS were investigated. Fourier transform infrared spectroscopy and hemispherical optical measurements in the solar range (0.3–15 μm) revealed that all nanocomposites exhibited enhanced emissivity within the 8–13 μm atmospheric window (ε_8–13_ ≈ 0.63–0.65) compared to pristine SBS, while the solar absorptance remained relatively low, although it increased with nanoparticle loading. By combining the experimentally determined emissivity and solar-weighted absorptance within a simplified energy-balance model, the radiative cooling potential of the films was assessed. Among the investigated formulations, 1NPs-SBS and 5NPs-SBS exhibited the most favorable balance between LWIR emissivity and solar absorption, highlighting the importance of nanoparticle loading optimization for passive radiative cooling applications.

## Graphical abstract

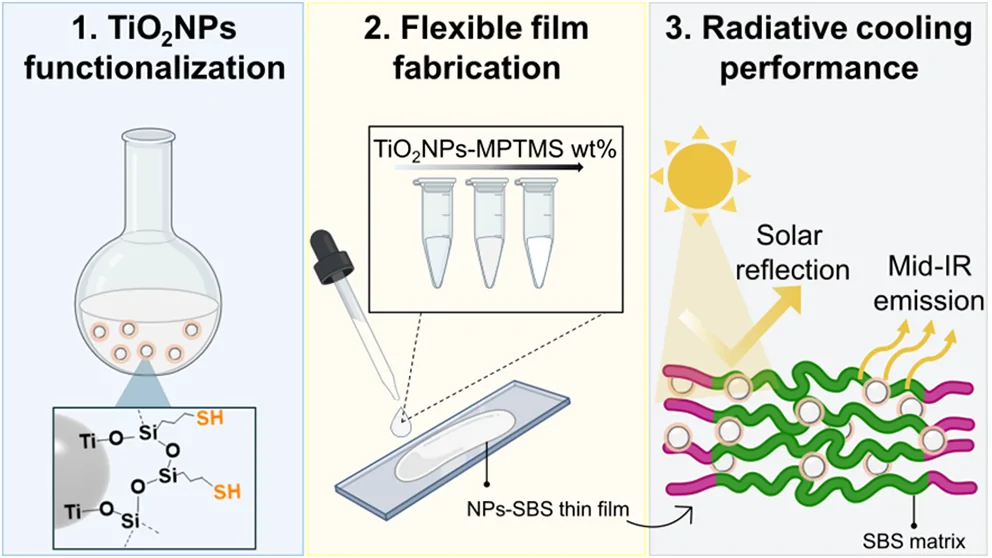

## 1 Introduction

Radiative cooling (RC) is a passive thermal management technique in which heat from terrestrial objects is dissipated as infrared thermal radiation into outer space, which acts as the ultimate heat sink with a temperature of approximately 3 K.^1,2^ This mechanism offers a promising strategy for mitigating global warming and climate change by providing cooling without any additional electricity consumption. RC is particularly suitable for applications such as cooling buildings,^3^ vehicles,^4^ outdoor equipment,^5^ and even the human body, providing a sustainable alternative to conventional active cooling systems such as HVAC (heating, ventilation, and air conditioning).^6,7^ Within the framework of passive RC, two infrared atmospheric transparency windows (AWs) are of critical importance: the midwave infrared (MWIR, 3–5 μm) and the long-wave infrared (LWIR, 8–13 μm) regions.^8^ These spectral windows allow thermal radiation to escape through the atmosphere without being absorbed by CO_2_ and water scattered in the atmosphere, in order to reach the external space.^6,9^ Conventional RC strategies primarily focus on the 8–13 μm AW, which aligns with the peak thermal emission of objects at ambient temperatures (∼300 K), according to Wien’s displacement law.^1,10^ On the other hand, the 3–5 μm window corresponds to thermal emission from hotter bodies (>600 K). This spectral region is particularly relevant for high-temperature components in aerospace, industrial furnaces, automotive and marine systems where efficient thermal emission and infrared signature suppression are critical.^10,11^

To meet growing demand for sustainable thermal management strategies, ongoing research is focused on designing new materials that exhibit high emissivity in the mid-infrared (MIR) range while also maintaining strong reflectance in the solar spectrum to obtain ideal radiative coolers.^2^ Among the wide range of candidate materials, polymers are especially promising in advancing RC technologies due to their intrinsic high IR emissivity, mechanical flexibility and ease of processing.^6^ Several polymer coatings have demonstrated excellent RC performance and are especially suitable for large-area applications such as paints, roof coatings, window films, and wearable cooling devices.^12,13^ The performance of polymer-based coolers can be further enhanced by incorporating ceramic micro- and nanoparticles as inorganic scatterers within the matrix.^14^ Moreover, the advantage of utilizing composite materials over complex photonic structures lies in their suitability for large-scale applications and cost-effective solutions. While photonic structures offer high spectral selectivity, their real applications are limited by complex fabrication.^15^ For instance, Park et al. embedded indium tin oxide (ITO) nanoparticles into a cellulose acetate polymer matrix, maximizing the IR emissivity and achieving a cooling effect of up to ∼2.7 °C under sunlight, thereby improving its cooling performance.^16^ Similarly, Hu et al. incorporated silicon dioxide nanoparticles (SiO_2_NPs) into a poly­(vinylidene fluoride-*co*-hexafluoropropylene) fiber matrix achieving superhydrophobicity, a remarkable solar reflectance of 98.5% and an average mid-infrared emissivity above 0.95.^17^ Recently, alternative strategies based on micro- and nanostructured polymeric architectures have also been proposed to enhance radiative cooling performance. For example, Yang et al. developed bioinspired macroporous polypropylene films containing randomly distributed three-dimensional pores, inspired by the structural whiteness of natural fibers. The engineered porous architecture promoted efficient solar backscattering, leading to solar reflectance values approaching 97% while maintaining significant LWIR thermal emittance. These results highlighted the importance of tailoring the morphology and scattering features of polymeric materials to simultaneously control solar reflectance and thermal emission, which are the two key requirements for efficient daytime radiative cooling.^18^

Among available fillers, titanium oxide nanoparticles (TiO_2_NPs) have attracted significant attention for RC applications due to their high refractive index and strong light-scattering capabilities. In both anatase and rutile forms, TiO_2_NPs are widely used as whitening pigments because of their ability to boost diffuse reflectance under visible and near-infrared (NIR) light, making them highly effective as solar reflectors.^19^ Although TiO_2_NPs can exhibit photocatalytic activity under UV illumination,^20,21^ they absorb predominantly in the UV-C (100–280 nm) and UV-B (280–315 nm) regions, which are largely filtered by the Earth’s atmosphere.^22^ Consequently, the risk of photodegradation in TiO_2_/polymer composites under outdoor conditions is substantially reduced. Embedding TiO_2_ micro/nanoparticles within a polymer matrix further reduces their available surface area and suppress their photocatalytic efficiency, thanks to a combination of high surface coverage through extensive intermolecular interactions, consistent with reports of excellent long-term stability in PDMS-TiO_2_ systems with negligible degradation.^23^ In addition to this, TiO_2_NPs possess a high refractive index, enabling efficient light scattering even at low pigment volume fractions, which makes them key components in solar-reflective roof coatings and radiative cooling materials.^24^ For instance, Nilsson et al. demonstrated that polyethylene foils pigmented with TiO_2_ and BaSO_4_ nanoparticles enhanced thermal emission and enabled atmospheric water condensation even under arid conditions, highlighting their potential for sustainable cooling technologies.^25^ Moreover, Bao et al. developed a double-layer nanoparticle-based coating composed of a top reflective layer of TiO_2_ particles and an underlying emissive layer made of SiO_2_ or Silicon Carbide (SiC) particles.^15^ The authors demonstrated that the application of double-layer coatings can significantly cool the original surface down especially at nighttime.^15^

Since IR radiation spans the entire atmospheric transparency window, the selection of materials for RC should consider not only their spectral reflectance but also the characteristic IR absorption bands of polymers (or molecules) related to the vibrational and rotational transition of their chemical bonds or functional groups. Polymers with chemical bonds such as C–C, C–O, C–F, C–Cl, and Si–O exhibit IR-active vibrational modes within the range 4000–400 cm^–1^, *i.e.*, 2.5–25 μm making them suitable for the preparation of effective radiative coolers.^6,26^ Furthermore, surface functionalization using an appropriate linker, such as (3-mercaptopropyl)­trimethoxysilane (MPTMS), is often required to improve the biocompatibility of the final product.^20^

In this work, a composite cooling film was developed by incorporating MPTMS-functionalized TiO_2_NPs into a styrene–butadiene–styrene (SBS) triblock copolymer matrix, using a previously optimized procedure^21^ that promotes the formation of Ti–O–Si chemical bonds at the nanoparticle–polymer interface, as shown in Figure 1. The resulting hybrid materials, denoted as NPs-SBS, were designed to combine enhanced solar reflectance with MIR emissivity. The SBS copolymer was selected due to its molecular bonds, such as C–H and C = C, which provide broadband IR absorption in the 10–12 μm range. In contrast to the rigid structures often reported in the literature, copolymers such as SBS exhibit inherent flexibility, allowing for their application on nonplanar and irregular surfaces. This mechanical adaptability is particularly advantageous for real-world and industrial applications, where materials are required to conform to complex geometries and diverse substrates. In parallel, the MPTMS organosilane shell surrounding the TiO_2_NPs core introduces additional vibrational modes, including Si–O, Si–C, Si–O–Ti, Si–O–Si, and C–H vibrations, covering the LWIR region. To assess the effect of nanoparticles concentration, freestanding composite films containing different amount of TiO_2_NPs–MPTMS at weight ratios of 1, 5, and 7% were incorporated into the SBS matrix to obtain freestanding composite thin film (namely, 1NPs-SBS, 5NPs-SBS, and 7NPs-SBS) to evaluate nighttime radiative cooling performance. This design has several advantages, including its high cooling capacity, simplicity, low cost, and ease of manufacture.

**Figure 1:**
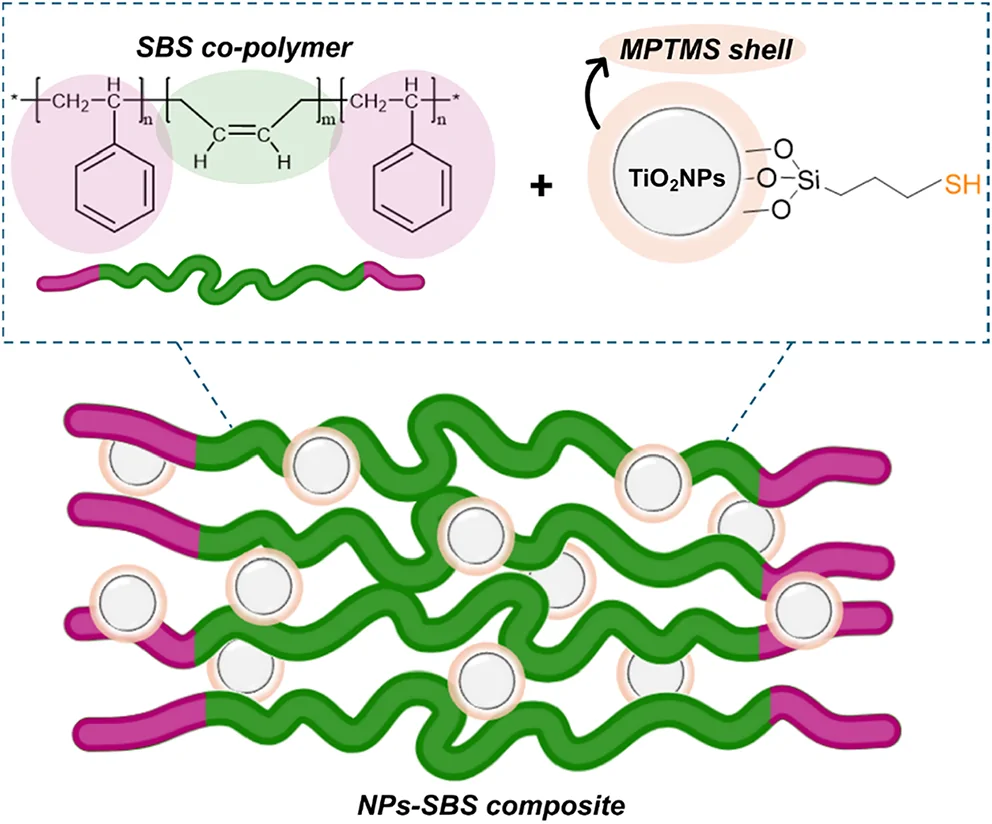
Schematic illustration of the NPs-SBS composite structure. The SBS triblock copolymer is composed of polybutadiene (green) and polystyrene (magenta) segments, forming a flexible matrix. TiO_2_NPs (white particles) are functionalized with an MPTMS shell (orange), which provides enhanced interfacial compatibility and introduces surface −SH groups. These functionalized TiO_2_NPs are physically embedded within the SBS matrix, enabling improved dispersion and MIR emissivity in the LWIR, relevant for nighttime RC.

The morphostructural properties were investigated by scanning electron microscopy (FESEM-EDS). Optical properties of the resulting composites were characterized at room temperature using Fourier transform infrared (FTIR) spectroscopy across the UV to MIR spectral range (0.3–15) μm. Compared to pristine SBS, all nanocomposites exhibited enhanced emissivity within the LWIR, highlighting the influence of the inorganic filler content on the thermal-radiative properties of the films. The RC potential of the investigated materials was assessed through a combined analysis of their emissivity in the AW and their optical response in the solar range (0.3–2.2 μm). Overall, the incorporation of MPTMS-functionalized TiO_2_NPs into the SBS matrix proved effective in tailoring the spectral emissivity of the films, making them promising candidates for passive RC applications.

## 2 Results and Discussion

### 2.1 Structural Design and Morphological Characterization

In this work, the SBS triblock copolymer was selected as the polymeric matrix for the fabrication of the NP-SBS composites. SBS is a thermoplastic elastomer known for its excellent thermal and mechanical stability, making it ideal for outdoor applications such as asphalt modification, energy devices, packaging, construction materials, and automotive interiors.^27^ SBS offers a distinct combination of optical and mechanical advantages, which is particularly relevant for building-scale applications.

Pure SBS is intrinsically opalescent-transparent; therefore, the progressive incorporation of TiO_2_NPs gradually increases its whiteness due to enhanced scattering in the visible range. This effect is beneficial as the material’s white visual appearance indicates stronger backscattering of solar radiation, resulting in reduced solar absorptance and improved cooling performance.^28,29^ In our system, SBS not only contributes to visible-light scattering when loaded with TiO_2_NPs but also acts as an effective encapsulation medium that immobilizes and protects the inorganic component within the polymer matrix, an aspect useful for ensuring durability in outdoor environments. In addition, compared to other polymers, such as PDMS (polydimethylsiloxane) and TPU (thermoplastic polyurethane), SBS can incorporate a high loading of inorganic fillers without losing flexibility or processability,^30^ allowing the fabrication of freestanding and bendable films that can conform to nonplanar surfaces, which is essential for building-scale applications and nanocomposite fabrication.

SBS-based thin films also display a significantly lower elastic modulus than PS (polystyrene)^31^ and PMMA^32^ films, resulting in improved adhesion to uneven or rough substrates useful for coating a variety of construction-related materials. Moreover, literature on PMMA–SBS blends demonstrates that the elastic modulus of PMMA drops sharply around 100 °C, while SBS remains mechanically stable up to at least 150 °C, maintaining its elastic properties during heating, showing much greater thermal mechanical stability.^32^ Its molecular structure features a polybutadiene segment alternating with polystyrene blocks, which contain unsaturated C = C bonds and aromatic rings, respectively (see Section 2.2).^33^ The styrene blocks form physical cross-linking points through π–π interactions between phenyl groups, while the butadiene phase provides elasticity to the matrix.^30^ These functional groups exhibit strong MIR emission, particularly between 10 and 12 μm, beneficial for RC applications.

To improve solar reflectance, the SBS matrix was combined with TiO_2_NPs since the solar reflectance of the polymer film can be well enhanced via the multiple Mie scattering induced by the porous micro- and nanoparticles, such as TiO_2_,^34^ TiO_2_/SiO_2_,^35^ and Al_2_O_3_ particles.^36^ These nanoparticles exhibit a high refractive index (*n* ≈ 2.5) and strong Mie scattering behavior in the visible to near-infrared (VIS-NIR) region, making them effective inorganic scatterers.^35,37^ In contrast, the refractive index of SBS lies in the range of 1.52–1.55, resulting in a significant optical contrast with TiO_2_NPs.^38^ As is well known, according to Snell’s law, large refractive index difference between two different media leads to a high magnitude of light refraction.^39^ This mismatch enhances multiple scattering events within the material, leading to greater solar light backscattering and overall increased reflectivity.

As previously reported,^35^ this scattering effect is critical to achieving ultralow solar absorption and boosting the thermal-radiative performance of cooling films. To ensure good dispersion of TiO_2_NPs within the SBS matrix and improve the compatibility between the inorganic and organic phases, the nanoparticles were surface-functionalized with MPTMS via a one-pot silanization process, as shown in Figure 2a, panel 1, and described in detail in Section 4.2. This functionalization leads to the formation of covalent Ti–O–Si bonds, resulting in a silica-like shell around the TiO_2_NPs.^21,40^ The resulting TiO_2_NPs–MPTMS not only exhibited improved colloidal stability within the polymer matrix but also introduce additional vibrational modes (Si–C, Si–O, Ti–O–Si), which further contribute to the overall infrared emissivity of the composite film. As a result, the strategic integration of MPTMS-functionalized TiO_2_NPs into the SBS matrix not only preserves the desirable properties of the polymer, such as processability, flexibility, and MIR emission, but also enables microstructural tuning of the composite to improve solar reflectivity. This synergistic design offers a cost-effective, scalable, and efficient strategy for developing high-performance RC materials. Subsequently, a solution of TiO_2_NPs–MPTMS and SBS copolymer in dichloromethane (DCM) was prepared as detailed in Section 4.3 (Figure 2a, panel 2). The mixture was then cast onto a clean glass slide; upon complete solvent evaporation, the freestanding composite films were detached from the substrate for optical characterization. The resulting composite films had a measured thickness in the 50–70 μm range. In the literature, the influence of thickness strongly depends on the optical design of the material. In some systems, reducing the film thickness has been shown to improve RC performance by modifying the scattering regime and enhancing spectral selectivity.^41^ In other cases, increasing the coating thickness enhances the effective optical path length and the amount of emissive bulk polymer, thereby improving the thermal emittance in the LWIR (ε_LWIR_).^6^ However, excessive thickness also increases material consumption and may compromise flexibility. Thus, it is essential to identify a suitable thickness that balances RC performance with practical considerations such as material cost and scalability.^6^ The 50–70 μm thickness obtained here represents a good compromise between mechanical robustness, film flexibility, and the optical requirements of our TiO_2_–SBS system.

**Figure 2:**
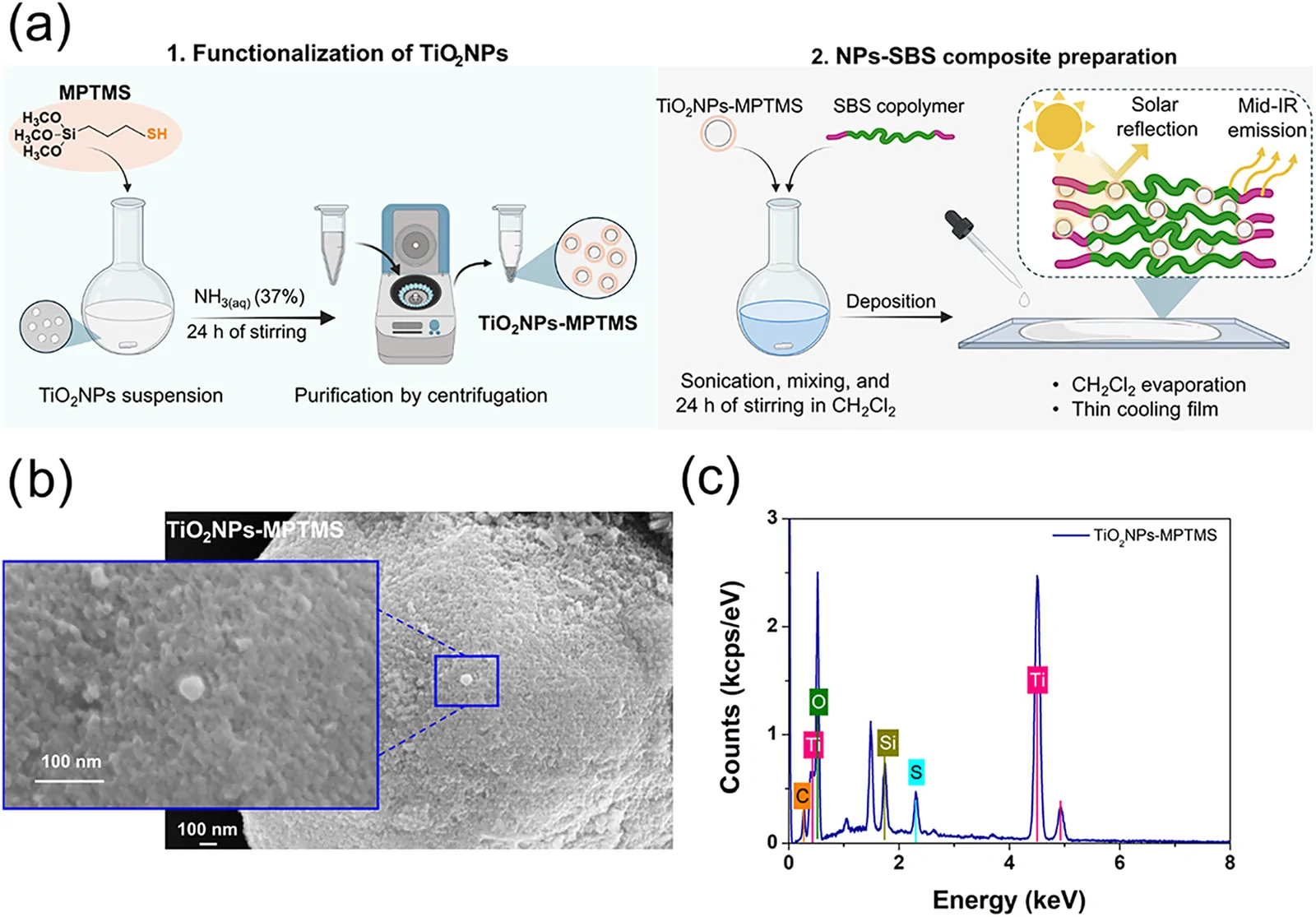
(a) Schematic illustration of the synthesis and fabrication process of the NPs-SBS composite thin films. (b) FESEM image of TiO_2_NPs–MPTMS. (c) EDS spectrum of TiO_2_NPs–MPTMS.

The solid-state morphology and size distribution of the functionalized nanoparticles were investigated using field-emission scanning electron microscopy (FESEM). As shown in Figure 2b, the TiO_2_NPs–MPTMS exhibit a grain-like morphology, forming macro-aggregates approximately 2 μm in diameter in the solid state. A closer look revealed that these aggregates were composed of smaller primary nanoparticles, with individual sizes predominantly in the 30–60 nm range. This hierarchical structure is particularly advantageous for RC applications, as the larger aggregates (in the range of 200–500 nm) can induce strong Mie scattering in the NIR region, effectively enhancing solar reflectance. The elemental analysis obtained through energy-dispersive X-ray spectroscopy (EDS), reported in Figure 2c, displayed distinct peaks corresponding to titanium (Ti), oxygen (O), silicon (Si), sulfur (S), and carbon (C). These elements confirm the successful surface functionalization of the TiO_2_NPs with MPTMS, and support the formation of a silica-based shell that improves chemical compatibility with the SBS polymer matrix.

### 2.2 Spectroscopic Characterization

#### 2.2.1 Transmission Analysis

FTIR spectroscopy is a powerful analytical technique for identifying the chemical composition of materials revealing structural information at the molecular level. Indeed, it is widely used to identify functional groups in organic compounds. The FTIR spectrum of pristine SBS (Figure 3a) exhibited the typical vibrations modes of the aromatic portions associated with the benzene rings in the SBS copolymer, and full assignments are reported in Table 1. These include the stretching (ν) of (=C–H) aromatic at 3006 cm^–1^, *in-plane* bending (δ) (=C–H) at 1034, together with *out-of-plane* δ­(=C–H) at 752 cm^–1^. Additional bands corresponding to the ν­(C–C) of the aromatic rings are observed at 1603, 1493, and 1451 cm^–1^. Deformations modes of benzene portion appeared at 843 cm^–1^. Vibrational bands at 2921 and 2846 cm^–1^ were due to asymmetric (ν_as_) and symmetric (ν_s_) stretching of −CH_2_ aliphatic chain of the butadiene segment, whereas ν­(C–H) and ν­(C = C) of polybutadiene block was found at 3061 and 1640 cm^–1^, respectively.^33,42–44^ Peaks at 966 and 699 cm^–1^ correspond to the characteristic absorptions of the cis-1,4 and *trans*-1,4 configurations of the butadiene units, respectively, indicating the presence of both configurations.^44^

**Figure 3:**
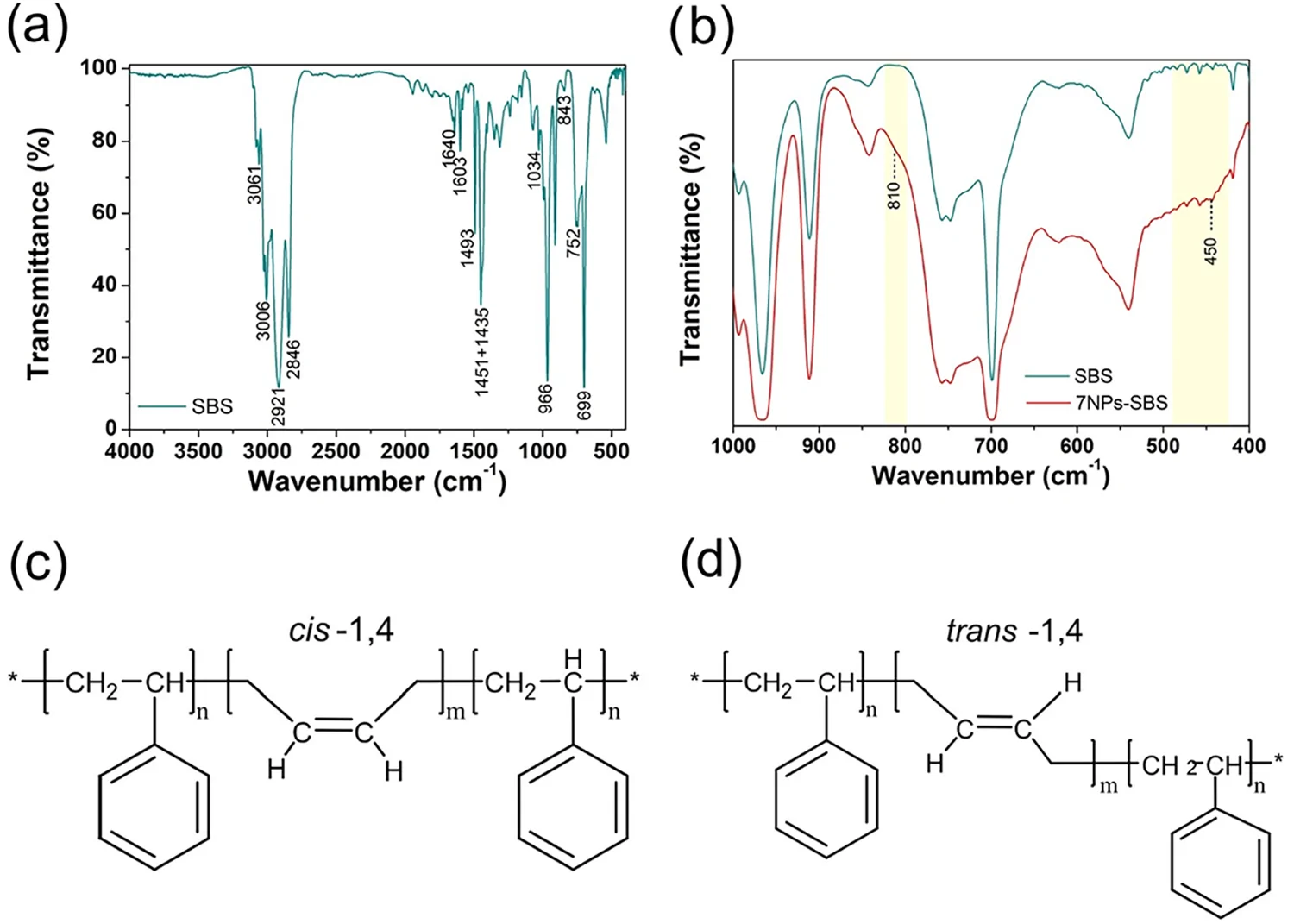
FTIR spectra in transmittance mode of (a) the pristine SBS film, (b) detail showing the pristine SBS film (green line) compared to 7NPs-SBS (red line) in the 1000–400 cm^–1^ region. Chemical structure of SBS copolymer with (c) *cis*-1,4 and (d) *trans*-1,4 configurations.

**Table 1:** FTIR Bands in the Mid-Infrared Region and Their Assignment for Pristine SBS^a^

assignment	wavenumber (cm^–1^) [refs 42–44 ]
ν(=C–H) polybutadiene	3061
ν(=CH) Ar	3006
ν_as_(−CH_2_)	2921
ν_s_(−CH_2_)	2846
ν(C = C) polybutadiene	1640
ν(C–C) Ar	1603
	1493
	1451
*in-plane* δ(=C–H)	1034
*trans* (=C–H)	966
deformation modes (C–C) Ar	843
*out-of-plane* δ(=C–H) Ar	752
*cis* (=C–H)	699

a Notation: ν—stretching; ν_s_—symmetric stretching; ν_as_—asymmetric stretching; δ—bending.

In Figure 3b, the FTIR spectrum of the 7NPs-SBS composite is shown as an example. The vibrational modes associated with the MPTMS linker are not easily detectable, likely due to the small amount of the stabilizing agent surrounding the TiON_2_NPs core and the spectral overlap with characteristic vibrations of the SBS polymer matrix. Indeed, the main SBS absorption bands, such as the C–H stretching vibrations around 2860–2930 cm^–1^, and the aromatic ring modes resulted to be very intense and broad in both spectra, effectively dominating the regions where the MPTMS signals would typically appear. The full merged spectra are reported in Figure S1a. Chemical structures of SBS copolymer with cis-1,4 and trans-1,4 configurations are reported in Figure 3c and d, respectively. However, the characteristic absorption band of Ti–O–Ti, embedded in the SBS matrix, is clearly detectable at 450 cm^–1^. Moreover, an additional absorption feature is observed at 810 cm^–1^ which can be attributed to the Si–C stretching vibration, as highlighted in yellow in Figure 3b.^21,45^ A similar spectral trend is observed for the 5NPs-SBS composite (Figure S1b). In contrast, for the 1NPs-SBS film containing the lowest nanoparticles concentration, the characteristic absorption bands associated with both Ti–O–Ti and the MPTMS linker are not evident in the spectrum, as shown in Figure S1. This suggests that the nanoparticle content in the matrix is too low compared to the organic counterpart, to produce detectable signals in the FTIR spectrum.

### 2.3 Optical Assessment of Radiative Cooling Performance

According to Kirchhoff’s law of thermal radiation, the emissivity of a body at a given temperature—defined as the ratio of its emitted radiation to that of an ideal blackbody—is equal to its absorbance under thermal equilibrium conditions.^7,46^ The optical response of the films was evaluated by considering both the MIR region, where high emissivity within the AW is desirable for RC, and the solar spectral range, where low emissivity is required to minimize solar heating. Neglecting reflection, which is a valid approximation in the case of rough, scattering films, the absorbance can be directly estimated from transmittance measurements as *A*(%) = 100 – *T*(%). The experimental curves reported in Figure 4b show that pristine SBS exhibits moderate emissivity within the LWIR range, with a few discrete bands. The incorporation of TiO_2_NPs into the SBS matrix leads to a significant improvement in the IR spectral region for all composite films (1NPs, 5NPs, and 7NPs, Figure S2), which is highly beneficial for RC applications. This enhancement can be attributed to the combined contribution of the SBS matrix, which inherently exhibits vibrational modes in the LWIR region, and the TiO_2_NPs–MPTMS component, which introduces additional vibrational features associated with Ti–O–Ti, Si–O–Ti, and Si–O–Si bonds. Overall, all nanocomposite films exhibit higher emissivity than pristine SBS within LWIR region, in line with literature observations on RC membranes incorporating TiO_2_NPs and Al_2_O_3_ microparticles into a PVDF-HFP polymer matrix.^37^ However, this improvement in the IR comes with a trade-off in the solar spectral range. Emittance spectra in the solar spectral range 0.3–2.2 μm (Figure 4a), calculated from hemispherical reflectance and transmittance measurements acquired with an integrating sphere (see Methods), show that the incorporation of 1 wt % TiO_2_NPs leads to higher emissivity in the visible region relative to pristine SBS. Although the incorporation of TiO_2_NPs enhances the IR emissivity and, consequently, the nighttime RC performance of the composites, the nanoparticle loading must be carefully optimized. Indeed, increasing the TiO_2_NPs content may simultaneously lead to higher solar absorption in the visible range (Figure S2), thereby partially offsetting the benefits of improved IR emission under daytime operating conditions.

**Figure 4:**
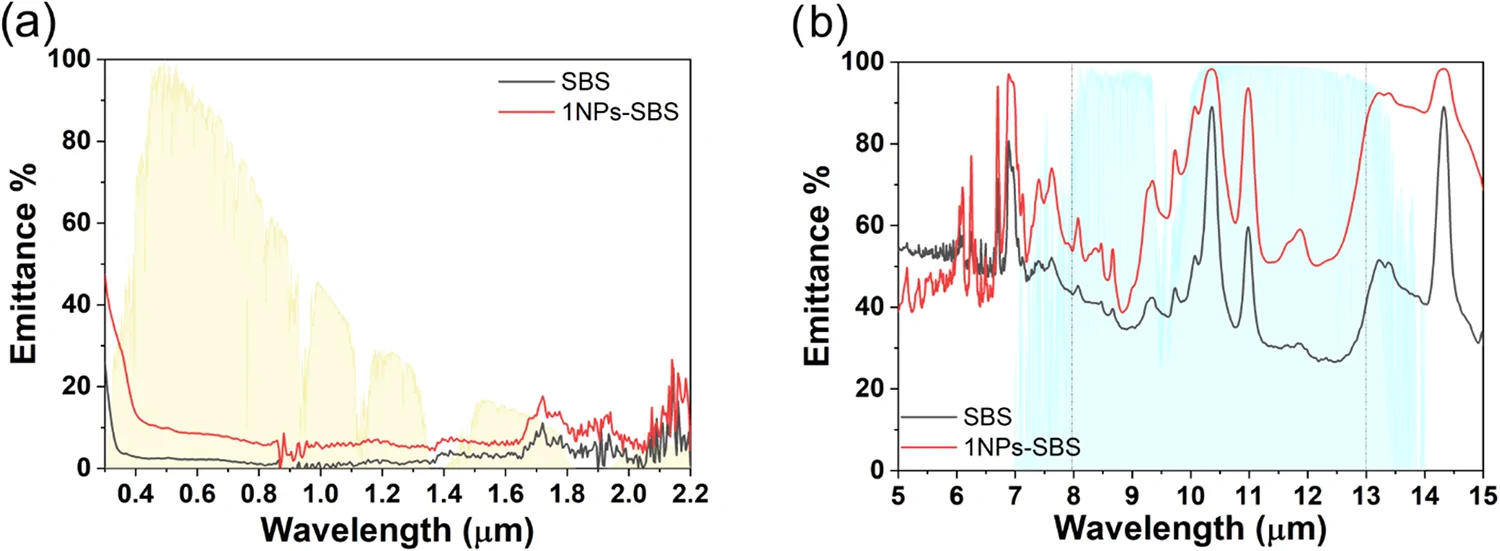
Optical properties of SBS and its related composites: (a) Emittance spectra in the solar spectral range (0.3–2.2 μm) derived from hemispherical reflectance and transmittance measurements using an integrating sphere, with the normalized AM1.5G solar spectrum plotted for reference; (b) IR emittance spectra in the 5–15 μm range, plotted alongside a realistic atmospheric transmittance model for reference. The dashed lines highlight the LWIR region, which is crucial for nighttime passive RC applications.

To compare the RC efficiency of different samples, their average emissivity ε_λ_min_–λ_max__(λ) was calculated within the LWIR region, for nighttime RC applications,^47^ using the following weighted integral (eq 1):

1 $$\varepsilon_{\lambda_{\min}-\lambda_{\max}}\left(\lambda \right)=\frac{\int_{\lambda_{\max}}^{\lambda_{\min}}B\left(\lambda \right)\varepsilon \left(\lambda \right)d\lambda }{\int_{\lambda_{\max}}^{\lambda_{\min}}B\left(\lambda \right)d\left(\lambda \right)}$$

where *ε*(λ) = α­(λ) = 1 – *T*(λ) is the spectral emissivity at different wavelengths which was retrieved from transmittance measurements. Similarly, *B*(λ) is the blackbody radiation calculated for a body at 300 K (room temperature) using Planck’s law $B\left(\lambda ,T\right)=\frac{2h_{p}c^{2}}{\lambda^{5}\left(e^{h_{p}c/\lambda k_{B}T}-1\right)}$ , where *h*_p_ and *k*_B_ are Planck’s and Boltzmann’s constants, respectively. This formulation allows the contribution of each wavelength to be appropriately weighted according to thermal emission intensity.

The behavior at shorter wavelengths becomes relevant when considering that solar radiation also contributes to the IR emissivity of the film. In order to evaluate the daytime devices’ efficiency, we introduce a parameter, *A*_VIS_ (λ), which considers the absorbance of solar-emitted radiation across the visible spectral range and it is calculated from the new integrating sphere measurements as α_VIS_ (λ) = 1 – *R*_hem_ (λ) – *T*_hem_ (λ) (eq 2):

$$A_{\mathrm{VIS}}=\frac{\int_{0.5}^{2.2}I_{\mathrm{solar}}\left(\lambda \right)\alpha_{\mathrm{VIS}}\left(\lambda \right)d\lambda }{\int_{0.5}^{2.2}I_{\mathrm{solar}}\left(\lambda \right)d\lambda }$$ 2

where *I*_solar_ (λ) is the global solar intensity and is calculated using Planck’s law for a body at 6000 K (Sun’s surface temperature). Pristine SBS exhibits relatively low average emissivity in the LWIR region, with a value of ε_8–13_ = 0.39 ± 0.14, mainly due to the discrete vibrational bands of the polymer backbone. Upon nanoparticle incorporation, the average emissivity increases markedly, reaching ε_8–13_ = 0.63 ± 0.01 for 1NPs-SBS, 0.63 ± 0.04 for 5NPs-SBS, and 0.65 ± 0.11 for 7NPs-SBS, as reported in Table 2. This enhancement indicates that the TiO_2_NPs–MPTMS phase contributes to broadening and strengthening the MIR absorption/emission response of the composite films within the AW. At the same time, the solar-range absorptance remains relatively low for all samples, although it increases with nanoparticle loading. Pristine SBS shows the lowest *A*_VIS_ value (0.02), while the nanocomposites exhibit values of 0.07, 0.05, and 0.12 for 1NPs-SBS, 5NPs-SBS, and 7NPs-SBS, respectively. This trend highlights the typical trade-off observed in RC-relevant materials:^35^ nanoparticle incorporation improves the MIR emissive response, but excessive loading may increase residual solar absorption.

**Table 2:** Summary of RC Parameters Derived from Emissivity and Solar Absorption Calculations (eqs 1 and 2) for SBS and NPs-SBS Composite Films^a^

sample	ε_8–13_	*A*_VIS_	Δ*T*
pristine SBS	0.39 ± 0.14	0.02	–2.57
1NPs-SBS	0.63 ± 0.01	0.07	–1.99
5NPs-SBS	0.63 ± 0.04	0.05	–3.16
7NPs-SBS	0.65 ± 0.11	0.12	0.71

a The *A*_VIS_ values were calculated from the hemispherical reflectance and transmittance spectra measured with the integrating sphere, using *α*_VIS_ (*λ*) = 1 – *R*_hem_ (λ) – *T*_hem_ (λ). The calculated Δ*T* (eq 3) compares performance across the LWIR region and also accounts for absorbed solar radiation (*A*_VIS_).

Figure 5 compares the spectral radiant power emitted by pristine SBS and 1NPs-SBS, calculated from their emissivity spectra using Planck’s law, with the theoretical blackbody emission at 300 K. The 1NPs-SBS sample exhibits higher emissivity within the LWIR window, confirming its enhanced suitability for nighttime RC. On the left side of the graph, the corresponding spectral radiant power is calculated at 6000 K (representative of solar radiation) for the two samples and for blackbody and appropriately scaled.

**Figure 5:**
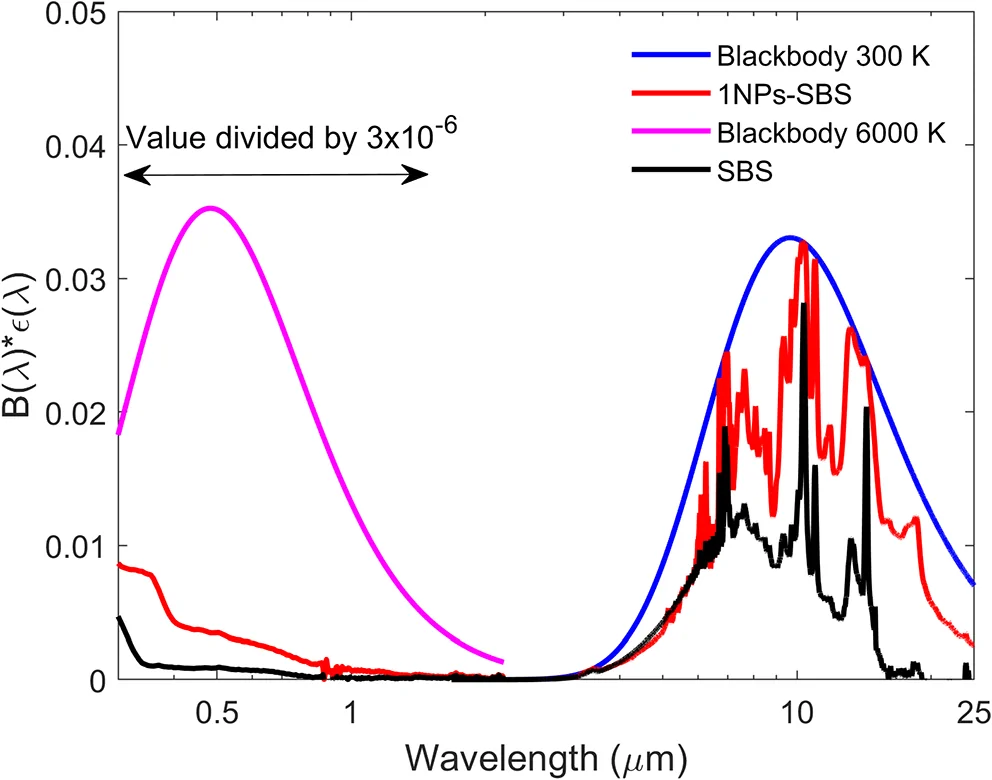
Comparison of the spectral emissivity between SBS (black line) and 1NPs-SBS (red line) films. The curves were obtained by normalizing the absorbance spectrum with blackbody radiation at 300 K (blue line) and 6000 K (magenta line), corresponding to the Earth and Sun temperatures, respectively. For clarity, the portion of the curves corresponding to the solar blackbody radiation (left side of the graph) was divided by a factor of 3 × 10^–6^.

Finally, to estimate the cooling capability of the investigated films, expressed as the temperature difference between the sample surface and the surrounding environment (Δ*T* = *T*_s_ – *T*_amb_), an energy-balance equation (per unit area) was adopted:

3 $$\varepsilon_{8-13}\cdot \sigma \left(T_{S}^{4}-T_{\mathrm{sky}}^{4}\right)+h\cdot \left(T_{s}-T_{\mathrm{amb}}\right)-\alpha_{\mathrm{VIS}}\cdot G=0$$

where σ denotes the Stefan–Boltzmann constant, *T*_s_ is the surface temperature of the material under consideration (to be calculated), *T*_amb_ is the ambient temperature, and *G* represents the incident solar irradiance. The radiative contribution was evaluated using the average emissivity ε_8–13_ measured within LWIR window, obtained from FTIR measurements, together with an effective sky temperature (*T*_sky_). The absorbed solar power (*A*_VIS_·*G*) was estimated from the solar-weighted absorbance *A*_VIS_, derived from the experimental optical spectra, and multiplied by the incident solar flux *G*. Convective heat exchange between the sample and the surrounding air was included through the term *h*·Δ*T*, where *h* is the effective convective heat-transfer coefficient. We selected as representative scenarios for daytime (*G* = 800 W/m^2^) while the effective heat-transfer coefficient *h* = 10 W·m^–2^·K^–1^. For temperature *T*_sky_, we used a value lower than ambient temperature (e.g., *T*_sky_ *= T*_amb_ *–*25 K for partially clear daytime). Samples exhibiting a lower *A*_VIS_ factor show negative Δ*T* values, indicative of net RC under representative conditions. In contrast, the sample 7NPs-SBS with the highest *A*_VIS_ yields a positive Δ*T*, confirming its reduced selectivity and limited effectiveness as a radiative cooler. This behavior can be explained by a recent theoretical study showing that the agglomeration of random micro-TiO_2_ particles embedded in a PDMS matrix strongly suppresses the solar reflectance of RC films.^48^ Wang et al. demonstrated that, at higher TiO_2_ volume fractions, particles tend to undergo agglomeration, thereby diminishing both the number of effective scattering centers and their individual scattering efficiency. This consequently leads to a pronounced decline in reflectance within the 0.4–2.5 μm solar range, i.e., the reflectance drops by 2–10%, significantly reducing cooling performance and increasing the equilibrium temperature of the film.^48^ In our system, the lower thermal response of the 7NPs-SBS films could be attributed to the same mechanism: higher NPs loading increases the probability of particle–particle contact and cluster formation, resulting in suboptimal solar scattering. Indeed, in particle-embedded radiative cooling films, the inorganic component predominantly governs the solar reflection properties, whereas the base polymer matrix largely dictates the emissive response in the LWIR window.^48^ Therefore, when particle agglomeration occurs, the reduction in effective scattering centers primarily affects the solar reflectance rather than the MIR emissivity, explaining why higher loadings (7 wt %) result in diminished cooling performance despite comparable LWIR emissivity. Consequently, the 1NPs-SBS composite film, in which the inorganic component could remain better dispersed, shows the highest solar reflectance and, therefore, the lowest apparent temperature in the indoor thermal experiment. Among the investigated samples, the 5NPs-SBS composite film exhibits the most balanced optical behavior, combining enhanced emissivity within the LWIR window with relatively low solar absorptance, which results in the lowest theoretical surface temperature.

## 3 Conclusions

In this work, flexible and freestanding nanocomposite thin films (1-, 5-, and 7NPs-SBS), based on styrene–butadiene–styrene (SBS) triblock copolymer and surface-functionalized titanium dioxide nanoparticles (TiO_2_NPs–MPTMS), were developed and characterized as scalable materials for passive nighttime RC. The TiO_2_NPs were functionalized with MPTMS via a sol–gel method conducted in a mixture of water/ethanol. This surface modification was crucial to ensure homogeneous dispersion within the polymer matrix, improve compatibility, and introduce additional vibrational modes aligned with the LWIR window, thereby enhancing MIR emissivity. Then, composites containing 1, 5, and 7 wt % TiO_2_NPs–MPTMS were prepared and optically characterized. FESEM–EDS investigations confirmed the intended multiscale morphology and successful surface functionalization, which improves compatibility with SBS matrix. Spectroscopic analysis across the UV–Vis/MIR range (0.3–15 μm) established that all NPs-SBS composites outperform pristine SBS within the LWIR window. In particular, the 1NPs-SBS and 5NPs-SBS formulations exhibited the most balanced optical behavior, combining high emissivity in the AW with limited solar absorptance. Overall, the proposed NPs-SBS nanocomposites represent a flexible, scalable, and low-cost platform for passive RC. The combination of enhanced emissivity in the AW and limited solar absorptance highlights their potential for thermal management applications, while also providing useful design guidelines for the development of future polymer-based RC materials.

## 4 Materials and Methods

### 4.1 Materials and Reagents

Titanium­(IV) dioxide nanoparticles (TiO_2_NPs) (anatase <25 nm, 99.7%), (3-mercaptopropyl)­trimethoxysilane [MPTMS: HS­(CH_2_)_3_Si­(OCH_3_)_3_], 95%, 196.34 g/mol, ethanol, ammonia (NH_3_), dichloromethane (DCM, CH_2_Cl_2_) were all purchased from Merck and used without further purification. Styrene–Butadiene–Styrene Block Copolymer (SBS, Europrene SOL T 6320) was purchased from Versalis (Eni group). Ultrapure Milli-Q water (H_2_O_up_, resistivity 18.3 MΩ·cm) was supplied by a Zeneer Power I Scholar-UV (Human Corporation) and used for the silanization of TiO_2_NPs.

### 4.2 Covalent Functionalization with MPTMS of Naked TiO_2_NPs

The silanization process of naked TiO_2_NPs was performed following a previously optimized procedure.^21^ In detail, 50.0 mg of commercial TiO_2_NPs were dispersed in a mixture of 20 mL of ethanol and 10 mL of ultrapure water (EtOH:H_2_O_up_, 2:1 v/v) and sonicated for 5 min. Subsequently, 15 μL of aqueous ammonia (NH_3(aq)_, 37%) was added as a catalyst to the TiO_2_NPs suspension, adjusting the pH to approximately 8. Then, 25 μL (25.0 mg) of MPTMS silane linker was introduced to the mixture, and the reaction proceeded for 24 h under continuous stirring. Subsequently, the MPTMS-coated TiO_2_NPs were purified by centrifugation with fresh H_2_O_up_ at 13,400 rpm, +8 °C, and 20 min (3×) to remove unreacted MPTMS and byproducts. The final product was freeze-dried to remove residual water and stored as a dry powder for subsequent use in the preparation of NPs-SBS films. The preparation process of silanized TiO_2_NPs is shown in Figure 2a.

### 4.3 Production of the Composite Material NPs-SBS

NPs-SBS composite films were fabricated using the following procedure: 50 mg of SBS copolymer was dissolved in 0.5 mL of DCM solvent and stirred using a magnetic stirrer until a homogeneous solution was obtained. Presynthesized TiO_2_NPs-MPTMS (see Section 4.2) were then dispersed in DCM at varying concentrations and added to the polymer matrix solution. Three composite formulations were prepared containing 1, 5, and 7% by weight of TiO_2_NPs-MPTMS, namely 1NPs-SBS, 5NPs-SBS, and 7NPs-SBS. For each film, the thickness was measured in five positions (in the center and at the sides), and then the average value was calculated. We report the mean ± standard deviation to quantify lateral uniformity which are reported below. The measured thicknesses are SBS (70 ± 8) μm, 1NPs-SBS (60 ± 14) μm, 5NPs-SBS (52 ± 3) μm, and 7NPs-SBS (57 ± 9) μm. These values indicate that thickness was consistent across different loadings, with variations primarily attributable to manual solution-casting rather than systematic changes induced by the nanoparticle content. In our NPs-SBS system, the selected thickness provides sufficient optical path length to obtain the observed emissivity enhancement (see Section 2.3). Each mixture was stirred for 24 h to ensure uniform nanoparticle distribution. The resulting suspensions were cast onto clean microscope slides, and the films were formed via DCM solvent evaporation at ambient conditions. After complete drying, the films were carefully detached to obtain freestanding NPs-SBS composite films and subsequently characterized in both the MIR and UV–Vis–NIR ranges (0.3–15 μm), as shown in Figure S2. The overall preparation process of the freestanding NPs-SBS cooling films is illustrated in Figure 2a, whereas pristine SBS and all the composites materials are shown in Figure S3.

### 4.4 Sample Characterization Techniques

The solid-state morphology and size of the nanoparticles were assessed by Auriga Zeiss field-emission scanning electron microscopy (FESEM) supported by an energy-dispersive X-ray spectroscopy (EDS) detector. The samples were drop-cast from their aqueous colloidal suspensions onto a conductive silicon substrate.

The MIR spectral features of several polymeric films were characterized and compared to evaluate their RC performance. FTIR measurements were carried out using a Bruker Invenio-R FTIR interferometer, operating across the spectral range of 4000–400 cm^–1^, or equivalently 2.5–25 μm. The interferometer utilized a glow-bar IR source and a deuterated triglycine sulfate (DTGS) pyroelectric detector. A total of 64 interferograms were acquired for each measurement, with a spectral resolution of 2 cm^–1^, selecting a sample area of 3 × 3 mm^2^ through window apertures.

The hemispherical measurements of the films at wavelengths ranging from 0.2 to 2.4 μm was performed using a PerkinElmer LAMBDA 1050+ UV/vis/NIR spectrophotometer equipped with a 2 Detector Module (WB) and with a 150 mm InGaAs Integrating Sphere.

## Supplementary Material



## Notes

FTIR spectra in transmittance mode for SBS and 7NPs-SBS in the 4000–400 cm^–1^ region; FTIR close-up transmittance spectra for pristine SBS, 1NPs-SBS, and 5NPs-SBS; emittance spectra in solar spectral range (0.3–2.4 μm) and mid-infrared range (5–15 μm) for pristine SBS, 1NPs-SBS, 5NPs-SBS, and 7NPs-SBS; and photographs of pristine SBS and NPs-SBS composite films (PDF)
