# Supplementary material for: Flexible Silanized TiO2/SBS Nanocomposite Films with Optimized Emissivity for Passive Radiative Cooling Applications
Source: ACS Appl Opt Mater. 2026 Jul 31;4(9):2698–707. doi: 10.1021/acsaom.6c00402 (PMC13622786; doi:10.1021/acsaom.6c00402)
Supplement: Supplementary file 1 [file ot6c00402_si_001.pdf]

Supporting Information

Flexible silanized TiO_2/SBS nanocomposite films with optimized emissivity for passive radiative cooling applications

Martina Mercurio[1*], Daniele Ceneda[1], Agostino Occhicone[1], Ilaria Fratoddi[2], Isabella Chiarotto[1], Marco Centini[1], Maria Cristina Larciprete[1]

[1]Department of Basic and Applied Sciences for Engineering, Sapienza University, Via Antonio Scarpa, 14, 00161, Rome, Italy

[2]Department of Chemistry, Sapienza University, P.le Aldo Moro 5, 00185, Rome, Italy

[*]Corresponding author:

Martina Mercurio (PhD, E-mail address: martina.mercurio@uniroma1.it)

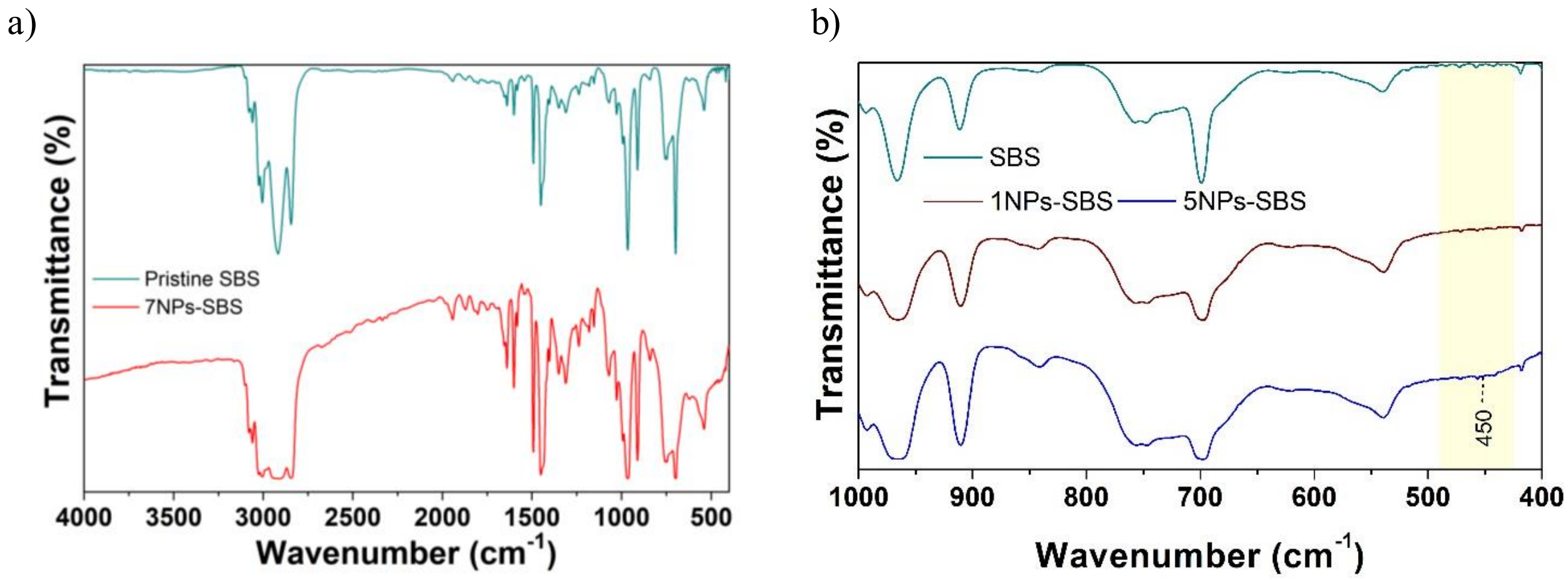

Figure S1. *FTIR spectra in transmittance mode for (a) pristine SBS film (dark cyan line) and 7NPs-SBS (red line) in (4000 – 400) cm^{-1} region. (b) SBS (dark cyan line), 1NPs-SBS (wine line), and 5NPs-SBS (blue line) films.*

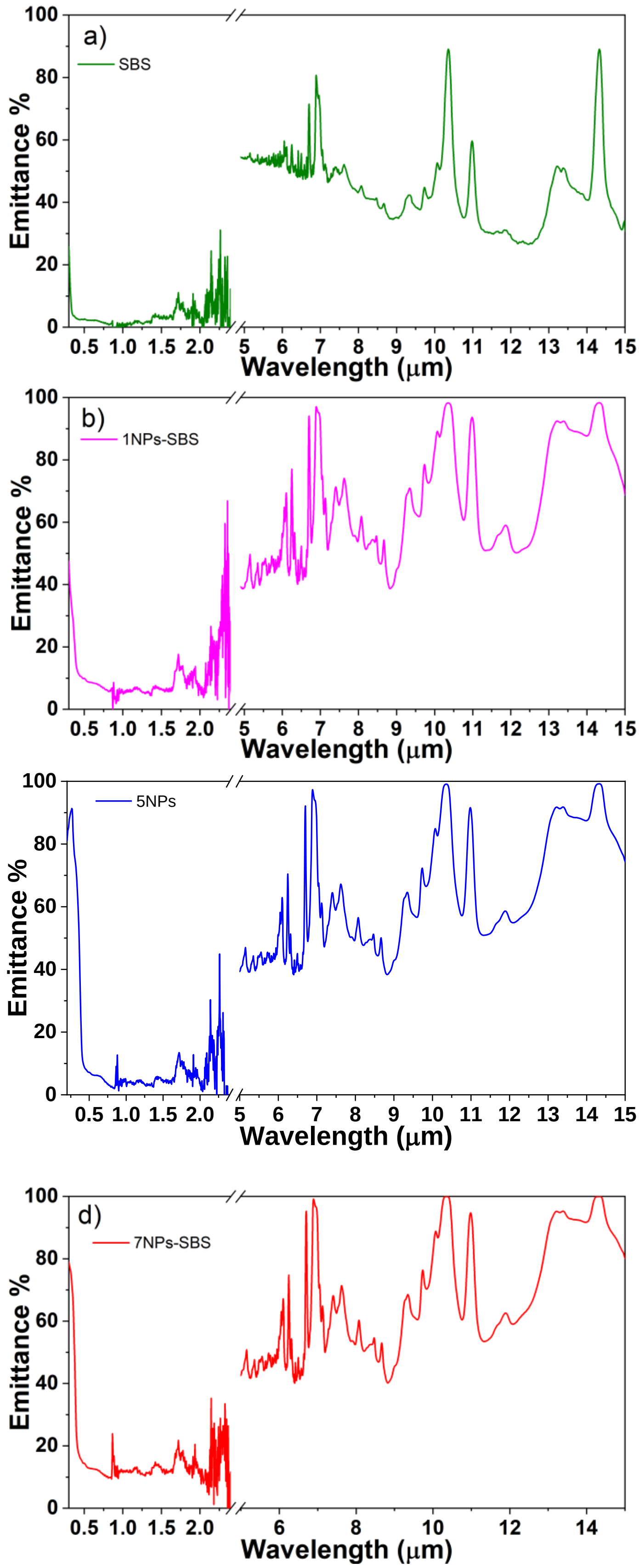

Figure S 2. *Emittance spectra in the solar spectral range (0.3–2.4 μm) derived from hemispherical reflectance and transmittance measurements using an integrating sphere and mid-infrared emittance spectra in the 5–15 μm range for (a) pristine SBS, (b) ~~SBS~~ 1NPs-SBS, (c) ~~SBS~~ 5NPs-SBS and (d) ~~SBS~~ 7NPs-SBS.*

Visually, the nanocomposite films vary from nearly transparent (pristine SBS) to progressively more opalescent and whitish with increasing TiO_2NPs-MPTMS loading. Notably, the 5NPs-SBS and 7NPs-SBS films exhibit a visibly whiter tint.

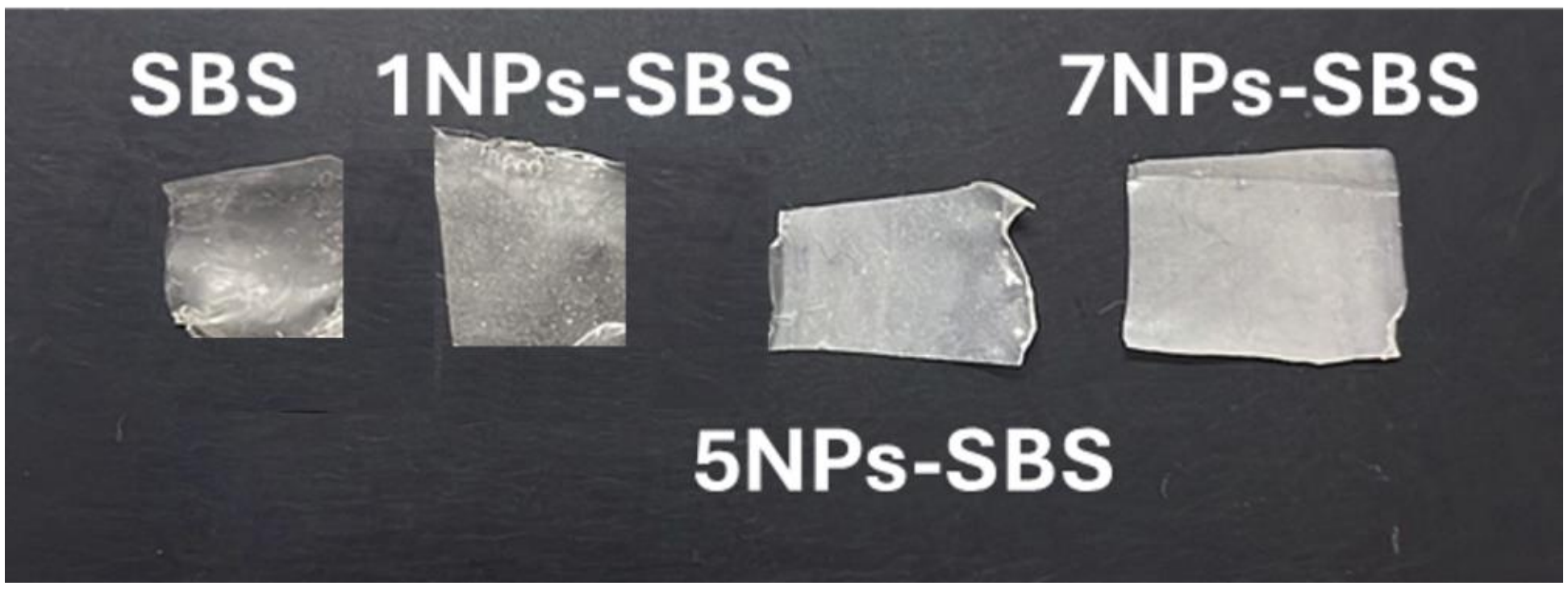

Figure S 3. *Photographs of pristine SBS and NPs-SBS films.*
